# Self-Mating in the Definitive Host Potentiates Clonal Outbreaks of the Apicomplexan Parasites *Sarcocystis neurona* and *Toxoplasma gondii*

**DOI:** 10.1371/journal.pgen.1001261

**Published:** 2010-12-23

**Authors:** Jered M. Wendte, Melissa A. Miller, Dyanna M. Lambourn, Spencer L. Magargal, David A. Jessup, Michael E. Grigg

**Affiliations:** 1 Molecular Parasitology Unit, Laboratory of Parasitic Diseases, National Institutes of Allergy and Infectious Diseases, National Institutes of Health, Bethesda, Maryland, United States of America; 2 Department of Veterinary Pathobiology, Oklahoma State University Center for Veterinary Health Sciences, Stillwater, Oklahoma, United States of America; 3 Howard Hughes Medical Institute–National Institutes of Health Research Scholars Program, Bethesda, Maryland, United States of America; 4 Marine Wildlife Veterinary Care and Research Center (CDFG-OSPR), Santa Cruz, California, United States of America; 5 Washington Department of Fish and Wildlife, Lakewood, Washington, United States of America; Duke University Medical Center, United States of America

## Abstract

Tissue-encysting coccidia, including *Toxoplasma gondii* and *Sarcocystis neurona*, are heterogamous parasites with sexual and asexual life stages in definitive and intermediate hosts, respectively. During its sexual life stage, *T. gondii* reproduces either by genetic out-crossing or via clonal amplification of a single strain through self-mating. Out-crossing has been experimentally verified as a potent mechanism capable of producing offspring possessing a range of adaptive and virulence potentials. In contrast, selfing and other life history traits, such as asexual expansion of tissue-cysts by oral transmission among intermediate hosts, have been proposed to explain the genetic basis for the clonal population structure of *T. gondii*. In this study, we investigated the contributing roles self-mating and sexual recombination play in nature to maintain clonal population structures and produce or expand parasite clones capable of causing disease epidemics for two tissue encysting parasites. We applied high-resolution genotyping against strains isolated from a *T. gondii* waterborne outbreak that caused symptomatic disease in 155 immune-competent people in Brazil and a *S. neurona* outbreak that resulted in a mass mortality event in Southern sea otters. In both cases, a single, genetically distinct clone was found infecting outbreak-exposed individuals. Furthermore, the *T. gondii* outbreak clone was one of several apparently recombinant progeny recovered from the local environment. Since oocysts or sporocysts were the infectious form implicated in each outbreak, the expansion of the epidemic clone can be explained by self-mating. The results also show that out-crossing preceded selfing to produce the virulent *T. gondii* clone. For the tissue encysting coccidia, self-mating exists as a key adaptation potentiating the epidemic expansion and transmission of newly emerged parasite clones that can profoundly shape parasite population genetic structures or cause devastating disease outbreaks.

## Author Summary

The parasites *Toxoplasma gondii* and *Sarcocystis neurona* have lifecycles that include a sexual stage in a definitive host and an asexual stage in intermediate hosts. For *T. gondii*, laboratory studies have demonstrated that the sexual stage can serve the dual purpose of producing new, virulent genotypes through recombination and promoting expansion of single clones via self-mating. Self-mating and other life history traits of *T. gondii*, including transmission of asexual stages among intermediate hosts, are assumed to account for the clonal population genetic structure of this organism. However, the relative contributions of sexual recombination and self-mating verses other life history traits in causing disease outbreaks or in shaping *Toxoplasma's* population genetic structure have not been verified in nature, nor have these traits been extensively examined in related parasites. To address this knowledge gap, we conducted population genetic analyses on *T. gondii* and *S. neurona* strains isolated from naturally occurring outbreaks affecting humans and sea otters, respectively. Our results identify self-mating as a key trait potentiating disease outbreaks through the rapid amplification of a single clone into millions of infectious units. Selfing is likely a key adaptation for enhancing transmission of recently emerged, recombinant clones and reshaping population genetic structures among the tissue-cyst coccidia.

## Introduction

Population genetic studies of pathogenic microbes have been paramount to our understanding of disease resulting from emerging and re-emerging infectious organisms [1]. Studies performed to determine the relative contributions of drift and recombination in the production of genetic diversity have identified that most pathogens have methods to alter, exchange and acquire genetic material that are intimately associated with pathogenicity [1], [2]. For viral pathogens, enhanced levels of drift, genomic reassortment [3], and incorporation of host genes [4] have all been linked to emergence of virulence. Likewise, horizontal gene transfer between bacterial species has facilitated assimilation of pathogenicity islands, plasmids, prophages, and other insertional elements essential for disease and drug resistance phenotypes [5]–[9]. For eukaryotic pathogens, meiotic sex serves an analogous purpose functioning to alter the genetic make-up, and therefore the biologic and virulence potential of strains [10]–[17]. A general paradigm describing disease epidemics for many pathogens is that genetic diversification, complemented by the acquisition of traits that enhance relative fitness and facilitate clonal expansion, leads to the emergence of novel, virulent genotypes. Just as the life history traits for generating genetic diversity vary widely among pathogen types, it is often the case that the mechanistic basis for subsequent clonal expansion of pathogenic strains is unique on a taxonomic level. Determining the mechanisms and contribution of these life history traits to disease is important for focusing prevention and treatment strategies to the most relevant pathogen strains and life cycle stages.

For the cyst-forming coccidia, which comprise a diverse group of parasites belonging to the phylum Apicomplexa, complex lifecycles that include both sexual and asexual stages have led to unusual population genetic structures for several species. For the widespread zoonotic pathogen, *Toxoplasma gondii*, the majority of strains infecting birds and mammals throughout North America and Europe are comprised of just three clonal lineages which exist as successful clones from a genetic out-cross [14], [18]. These three lineages have apparently emerged only recently due to an enhanced fitness that facilitated their ability to effectively outcompete other genotypes [13], [19]–[21]. Likewise, the veterinary pathogen *Sarcocystis neurona* possesses a surprisingly simple population genetic structure punctuated by the dominance of a few clonal lines in North America [22]–[25]. Similar clonal structures have been reported for other parasitic protozoa that possess sexual cycles [26] but identifying the precise genetic mechanisms that have led to the emergence of distinct clones among the different species in nature remains enigmatic.

In combination with population genetic data, the contributions of sexual out-crossing and clonal expansion as factors governing the emergence and eventual dominance of distinct, disease-producing clones have largely been inferred from laboratory studies of *T. gondii* among the cyst forming coccidia. Prior experiments demonstrated that a sexual cross between mouse-avirulent strains can produce genotypes representing a range of virulence in the mouse model, including some progeny several logs more virulent than the parents [14]. This study identified that natural out-crosses likely produce at least some virulent genotypes, which may subsequently have potential to emerge through clonal amplification to cause extensive disease [20], [21]. Clonal propagation is possible since *T. gondii* can effectively bypass the sexual stage in felid definitive hosts and cycle, presumably indefinitely, among intermediate hosts. This can occur horizontally via oral transmission through carnivory among intermediate hosts [19], [20] or vertically by transplacental transmission [27]–[30]. *Toxoplasma gondii* can also functionally bypass genetic diversification during the sexual stage by self-mating in the definitive host. Self-mating (also termed selfing, uni-parental mating, or self-fertilization) occurs when a single parasite clone can give rise to both male and female gametes capable of undergoing fertilization and producing viable offspring [31], [32]. In other words, no predetermined mating types are apparent and the end result is effectively clonal expansion via sex and meiosis.

Despite these important laboratory studies, the implications of these life-history traits and their relative effects on population genetic structures, especially in the context of virulence and disease outbreaks, have not been extensively studied in *T. gondii* or other cyst forming coccidia in a natural setting. Parasite life stages that are most important for causing mass-morbidity and mortality may be revealed through review of past, large-scale *T. gondii*-associated human outbreaks. For eleven reports of *T. gondii*-associated disease outbreaks in immune-competent people, eight events, including the four most devastating that caused disease or death in hundreds of individuals, were attributed to the oocyst form of the parasite, which is only produced during the sexual life cycle stage in the definitive feline host [20]. Furthermore, an outbreak of the related veterinary pathogen *Sarcocystis neurona* that resulted in the death of nearly 1.5% of the threatened Southern sea otter population over the course of a single month is thought to have resulted from exposure to infectious sporocysts originating in the definitive opossum host [33]. Circumstantial evidence, such as a complete lack [34] or much reduced [35]–[37] prevalence of *T. gondii* in certain island environments without cats, also gives weight to the importance of the definitive host stage in the parasite life cycle. Similarly, *S. neurona* has not been identified outside of its definitive host range in the Americas. The apparently profound importance of this stage in the lifecycle of not just *T. gondii*, but other related parasites, warrants further study to determine the influence it could impart to shaping parasite population genetic structures and which genetic mechanisms inherent to this life stage (i.e. selfing or out-crossing) are more likely to precede a disease outbreak in nature.

To determine the genetic basis governing the exposure, evolution, and emergence of virulent genotypes during natural outbreaks linked to sexual stages of these parasitic protozoa, we tested whether epidemic isolates exist as: 1. a diverse array of multiple, novel genotypes that are the products of an out-crossing event in the definitive host, or 2. epidemic clones of a single genotype derived via selfing in the definitive host. To distinguish between these two possibilities, high resolution genetic typing was used to characterize parasite strains associated with a *T. gondii* outbreak in humans [38] and a *S. neurona* outbreak in sea otters [33], both of which were associated with unusually high levels of morbidity and mortality. The population level genetic studies presented here argue that selfing in the definitive host plays a central role in the epidemic expansion of newly emerged, recombinant parasite strains, thus potentiating clonal outbreaks caused by tissue cyst-forming coccidia.

## Results/Discussion

### An outbreak linked to *T. gondii* oocyst ingestion was associated with a single parasite genotype

**Figure 1:**
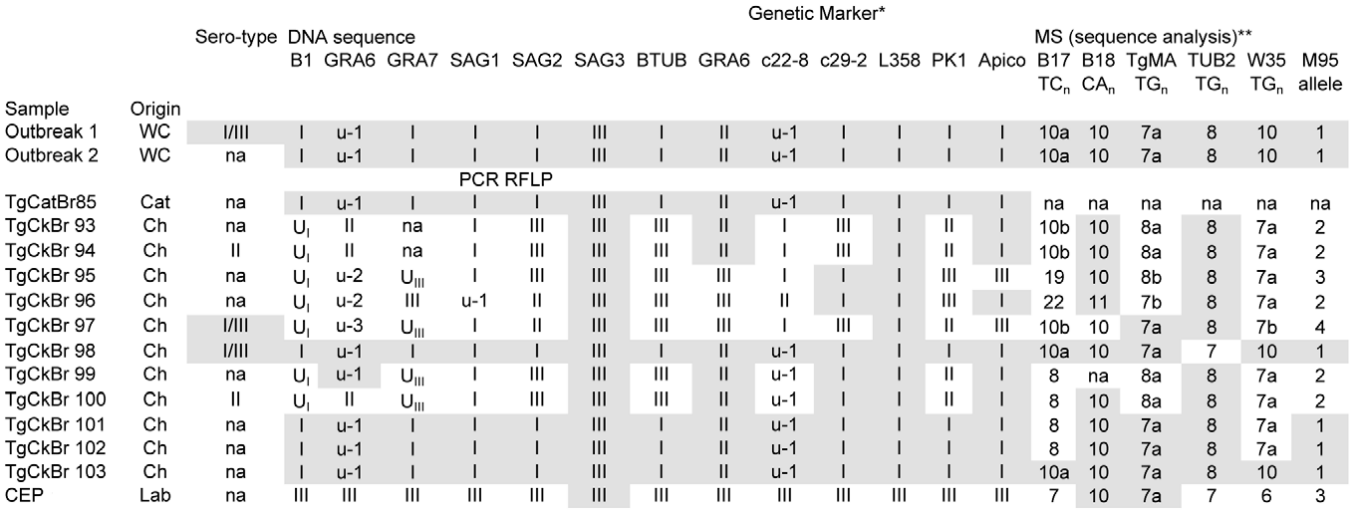
Genotype analysis of *Toxoplasma gondii* strains associated with an outbreak in Santa Isabel do Ivai, Brazil. All *T. gondii* isolates were analyzed directly by sequencing at microsatellite (MS) loci and PCR-RFLP at the remaining loci except for Outbreak 1 and Outbreak 2 which were directly sequenced at all loci. Outbreak 1, Outbreak 2, TgCatBr85, and TgCkBr98–103 all possess one of two alleles at each locus, suggesting they are sibling progeny from a recent outcross. Outbreak 1 and Outbreak 2 were oocyst samples isolated from two separate water filters from water supplies implicated in the outbreak and possess identical genotypes indicative of a clonal outbreak. This suggests an outcross preceded the outbreak and was followed by a selfing event in the definitive host that enhanced the clonal expansion and transmission of the newly emerged, recombinant outbreak genotype. Shaded alleles indicate those which are identical to the Outbreak genotype. *Serotype, DNA sequence, and PCR RFLP data from Vaudaux et al. [44]; **Numbers indicate dinucleotide repeat count and letters indicate distinguishing SNPs surrounding the repeat region; MS: microsatellite; WC: water cistern; Ch: chicken; Lab: laboratory strain; na: not available.

A microsatellite-based typing scheme using the markers B17, B18, TgMa, TUB2, W35 [39], and M95 [40] was applied to determine the molecular genotypes of *T. gondii* isolates associated with a human water-borne outbreak in Brazil. This outbreak, which occurred over a short time span in 2001, was linked to oocyst-contamination of a municipal water supply in the town of Santa Isabel do Ivai and resulted in infection and symptomatic disease in hundreds of people [38]. Initial genetic typing analyses performed on two *T. gondii* strains isolated from the water cistern implicated as the source of the outbreak [38], as well as isolates from chickens [41] and cats [42] from the immediate environment were limited to PCR-RFLP at a single locus, SAG2, leading to the conclusion that the outbreak strain was a canonical Type I strain (see below). Later, more extensive analysis by PCR-RFLP [43] and DNA sequencing on a limited set of markers [44] showed that the outbreak-associated strains from the water cistern were clonal and non-archetypal. The majority of people who seroconverted during the outbreak also possessed a serologic profile consistent with infection by the outbreak clone, and the outbreak genotype appeared to be highly prevalent in the surrounding environment immediately following the outbreak event, infecting 4/11 chickens (TgBrCk98, TgBrCk101–103) and 1 cat (TgCatBr85) [44] (Figure 1).

To determine the extent of genetic relatedness among the outbreak-associated strains, high resolution MS typing and DNA sequencing using markers distributed on 11 of the 14 chromosomes was applied. This dataset distinguished the two water cistern, outbreak-associated strains at the genetic level from all others present in the environment, except for one chicken isolate (TgCkBr103) (Figure 1). Unfortunately, insufficient DNA remained from the cat isolate, TgCatBr85, which precluded testing whether it was genetically identical to the cistern isolates.

Utilizing the MS typing scheme confirmed the conclusion that the causal agent was a unique, emergent *T. gondii* strain with a potential for enhanced virulence. The additional typing provided in the current study refined the conclusions of previous studies in two key aspects. First, the much higher level of resolution provided by the markers used and the sequence level analysis imparts a higher level of confidence to the conclusion that the outbreak was in fact clonal. The possibility that the outbreak-associated clones are not genetically identical in lieu of additional typing cannot be excluded, but several facts strongly argue against this: 1. The 18 markers were distributed across all but three of the 14 chromosomes; 2. MS markers are prone to rapid evolution and therefore provide high resolution; 3. Strains from Brazil are genetically divergent from archetypal lines, as evidenced by the segregation of alleles amongst strains in Figure 1, and hence, less prone to linkage disequilibrium effects. Furthermore, only a single, oocyst-derived clonotype was isolated from independent filters collected from two different water-holding tanks providing additional evidence that these isolates resulted from self-mating rather than a genetic out-cross.

Second, this study refines previous work on the Santa Isabel outbreak by showing that the outbreak strain was actually rare in the surrounding environment, opposed to the high prevalence reported previously [44]. Moreover, close examination of the environmental isolates reveals that many of them, including those previously identified as the outbreak clone, and the outbreak clone itself, resemble recombinant progeny; only two allelic types are present that segregate independently across the loci examined (see TgCkBr98, 99, 100, 101, 102, 103, TgCatBr85 and Outbreak 1 and 2 in Figure 1). These data argue that prior to the outbreak, the epidemic clone was produced by a genetic out-cross and was subsequently expanded by self-mating. This confirms that the more extensive resolution provided by the current study was necessary to truly distinguish an epidemic clone in a region known to contain a diverse array of *T. gondii* genotypes, including many that are apparently siblings of this strain [44]. This result also speaks to the important role selfing in the definitive host can play; allowing a single, emergent genotype of low environmental prevalence to rapidly rise to dominance in the surrounding population by infecting several hundreds of hosts over a short time span.

Collectively these data support high-resolution genotyping schemes as important tools for detecting informative genetic signatures in this parasite species. Initial population genetic studies showed that *T. gondii* strain diversity was comprised of three main clonal groups: Type I, II, and III [18]. As a result of these early studies, many broader population genetic studies have since relied on typing at only one or just a few loci to classify strains as type I, II, or III. However, it is now apparent that strains from diverse geographic locales and host species are more often infected with strains bearing unique alleles or allelic combinations, so relying on a few markers is insufficient for robust conclusions [20]. The first quantitative analysis testing the accuracy of single locus typing found a very low predictive value for the loci analyzed to correctly identify strain genotype [45]. Indeed, results presented in the current study, when compared with results from more limited genetic studies of the same strains conducted previously [38], [41]–[44], provide a clear illustration of the value more extensive genetic typing can have in refining conclusions. This is especially relevant in outbreak investigations where variations in parasite genotype can be highly informative for explaining disease manifestation. High-resolution genetic typing appears to be critical for eliminating preconceived biases in epidemiologic investigations to ensure accurate discernment of disease-associated *T. gondii* strains and to recognize clonal outbreaks.

These results validate the utility of testing for epidemic clones from prospective and retrospective studies of *T. gondii* disease outbreaks [20]. In support of this, Dumar and colleagues applied a similar typing scheme to a *T. gondii* outbreak in Suriname and discovered that all five patients from whom they isolated parasites were infected with the same, previously undiscovered genotype [46]. Importantly, the outbreak in Suriname was another waterborne outbreak attributable to human exposure by infectious oocysts, further evidencing selfing in the definitive host as a key mechanism for allowing clonal expansion of virulent genotypes, ultimately resulting in disease epidemics.

### Genetic typing of outbreak strains of the related pathogen, *Sarcocystis neurona*

Since parasite genetic material from past *T. gondii* outbreaks in humans is in limited supply for the majority of cases, we sought to further assess the role of self-mating in disease outbreaks by examining an epizootic of the related veterinary pathogen, *Sarcocystis neurona*, infecting the Southern sea otter (*Enhydra lutris nereis*) of California. As a threatened species, the Southern sea otter population is well monitored and accounted for by conservation groups, creating a unique opportunity to investigate infectious disease in a natural setting. Sea otters are also aberrant hosts for many terrestrial pathogens that can be washed to sea and their high susceptibility to many of these pathogens allows them to serve as a sentinel species for pathogens circulating in the adjacent terrestrial environment [44]. During April, 2004, the highest monthly mortality rate ever recorded in nearly 30 years of data collection occurred among Southern sea otters [33]. Over the course of approximately one month, at least 40 sea otters stranded dead or dying along an 18 kilometer stretch of coast within the 500–600 kilometer Southern sea otter range. Sixteen otters were in sufficient condition to allow for complete post-mortem analysis inclusive of PCR assessment and microscopic examination of tissues. Among these otters, the major cause of death for 15 of the 16 examined animals was *S. neurona*-associated brain and/or systemic disease [33].

Preliminary genetic analysis using only four polymorphic markers against parasite strains infecting a subset of these otters (n = 7) suggested they were genetically homogenous [25]. However, the limited polymorphism present in the markers used, and lack of information about the population genetic structure of *S. neurona* in California prevented a confident conclusion that they represented an epidemic clone. The present study developed and applied a battery of higher resolution, polymorphic microsatellite and gene-coding markers to type *S. neurona* strains. Additional samples were included, encompassing 12 *S. neurona* strains from otters that died during the outbreak, as well as additional strains from other geographic locations and/or time periods. The high number of sea otter deaths associated with this epizootic provided a unique opportunity to test whether self-mating, as identified in the human *T. gondii* outbreaks, could explain the genetic origin for the *S. neurona* strains that caused the outbreak. In addition, genetic data from the current study was combined with *S. neurona* typing data reported by Rejmanek et al. [23] to determine the population genetic structure of *S. neurona* in California spanning 15 years of study.

### eBURST analysis reveals two main *S. neurona* clonal complexes in California

**Figure 2:**
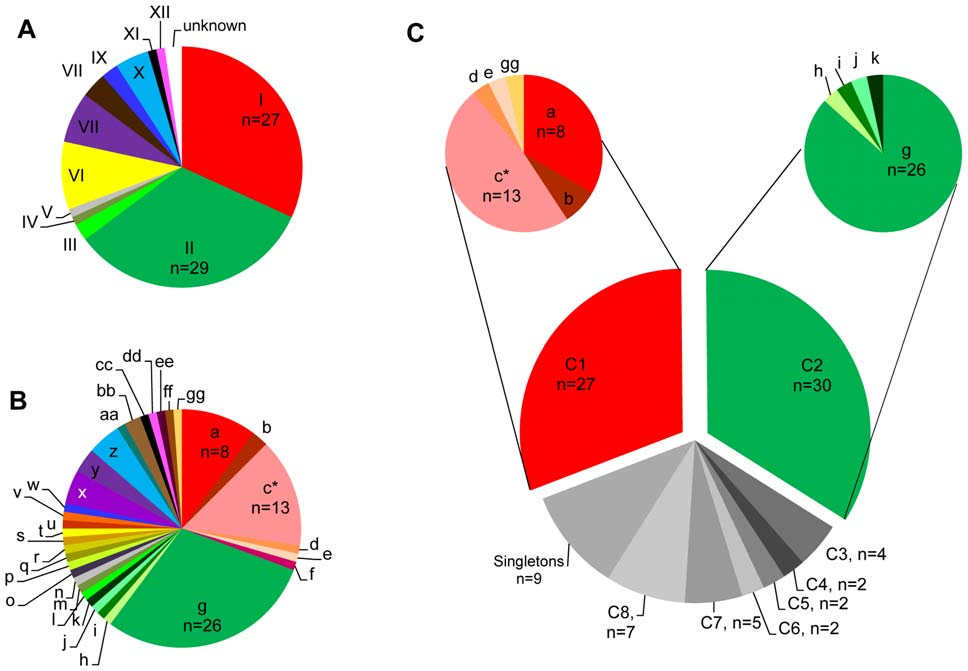
*Sarocystis neurona* genotyping results. Distribution of the 12 Ag types (A) and 33 MS types (B) identified among all *Sarcocystis neurona* samples studied (n = 87). Ag type I and II accounted for the majority of all samples with 27 and 29 samples, respectively. The most numerous MS type identified was type g, accounting for 26 total samples. (C) Further analysis of MS types using the eBURST program on default settings for 9 loci (Sn2–Sn5, Sn7–Sn11), revealed that 64% of all isolates belonged to two clonal complexes. Clonal complex 1 (CC1) was comprised of MS types a, b, c, d, e, and gg and CC2 of types g, h, i, j, and k. All MS types in CC1 possessed Ag type I. MS types g, h, i, and j of CC2 possessed Ag type II, whereas MS type k possessed Ag type III. *MS type c was found in 11/12 examined *S. neurona* strains from sea otters that died during the 2004 epizootic.

Sequence-level analysis of five surface antigen (Ag) genes (SnSAG1, 3, 4, 5, and 6) [25] and nine microsatellite (MS) markers (Sn2–Sn5, Sn7–Sn11) [23], [25] identified 12 Ag types and 33 MS types among 87 *S. neurona*-infected samples based on the allele combinations detected at each locus (Table 1; See Table S1 for complete strain and typing information). Seventy-four of the 87 samples were from mammals in California; other states represented include Georgia (n = 2), Illinois (n = 1), Missouri (n = 3), Washington (n = 5), and Wisconsin (n = 2). Combining Ag and MS alleles could distinguish 35 total genotypes, but for this study these typing schemes were analyzed independently because of the likelihood that these parts of the genome are under different selection pressures and subject to differing evolutionary processes [2]. The majority (56/87) of *S. neurona* strains were classified as either Ag type I or Ag type II (Figure 2A). Certain MS types were also over represented in the sample set, with MS types ‘a’, ‘c’, and ‘g’ accounting for 47/87 samples (Figure 2B). Importantly, 11/12 *S. neurona* strains from sea otters stranding during the mortality event in 2004 were an exact genetic clone at each marker analyzed (Ag type I, MS type ‘c’). The remaining outbreak sample (Ag type I, MS type ‘d’) differed from the other outbreak strains by only a single stepwise mutation at MS marker Sn4 (Table S1).

**Table 1:** *Sarcocystis neurona* genotyping data summary.

Data Set	Host	Samples	Antigen types	MS types	total genotypes	eBurst complexes (MS)	eBurst singletons (MS)	Proportion complex 1	Proportion complex 2	Total proportion complex 1/2
Overall	Sea otter	57	9	20	20	5	2	0.47	0.32	0.79
	Harbor seal	6	2	6	6	nd	nd	0	0.17	0.17
	Raccoon	2	1	2	2	nd	nd	0	0	0
	Opossum	13	6	7	9	1	5	0	0.31	0.31
	Horse	7	3	4	4	nd	nd	0	0.57	0.57
	Porpoise	1	1	1	1	nd	nd	0	1.00	1.00
	Cat	1	1	1	1	nd	nd	0	1.00	1.00
	Total	87	12	33	35	8	8	0.31	0.34	0.65
Monterey, CA (ATOS 1–400)	Sea otter	30	9	12	12	4	2	0.07	0.63	0.70
	Opossum	10	5	5	6	1	3	0	0.40	0.40
	Horse	4	2	2	2	nd	nd	0	0.75	0.75
	Porpoise	1	1	1	1	nd	nd	0	1	1.00
	Total	45	11	15	16	4	4	0.07	0.60	0.64
Morro Bay, CA (ATOS 800–1200)	Sea otter	27	3	9	9	1	3	0.93	0	0.93
	Total	27	3	9	9	1	3	0.93	0	0.93

MS: microsatellite.

nd: not done.

ATOS: The ‘As The Otter Swims’ number refers to each sea otter's stranding location, based upon defined and sequential 0.5 kilometer segments of the California coastline, starting with zero (0) just north of San Francisco and increasing numerically from north to south.

**Figure 3:**
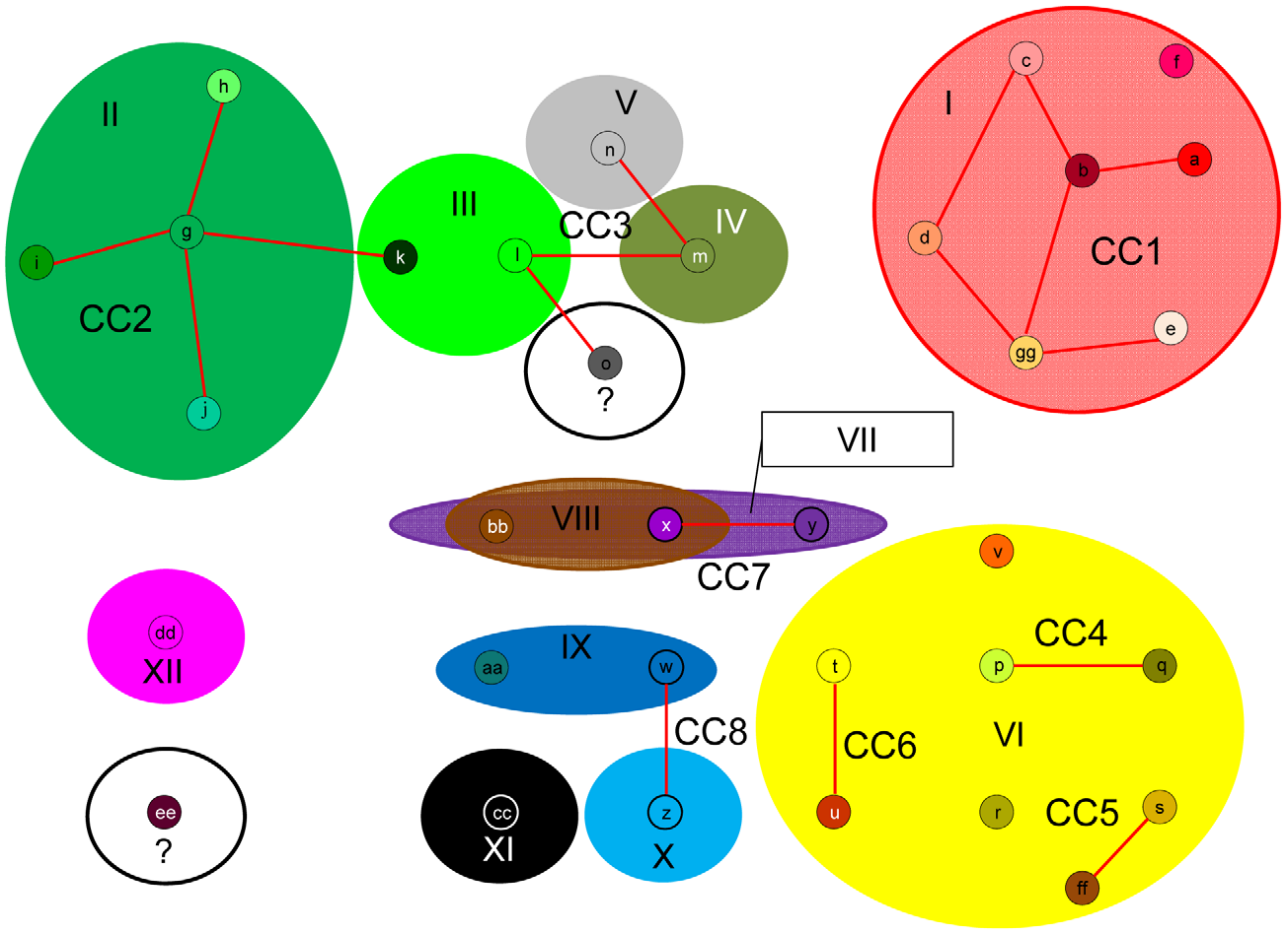
Modified eBURST analysis output. Default eBURST settings were used to analyze *Sarcocystis neurona* sequence types based on MS markers Sn2–Sn5 and Sn7–Sn11. MS types identified are represented as small circles and designated by lowercase letters. Lines connect MS types that are identical at 8 out of 9 MS loci and are therefore considered part of a clonal complex (CC). eBURST identified 8 clonal complexes (designated CC1–CC8) and 8 singletons. Large colored ovals are overlain to indicate the Ag type (Ag types I–XII) that characterizes each MS type identified by eBURST. MS and Ag type color schemes refer to those described in Table S1. Results support an intermediate population structure with both clonal propagation and sexual recombination. All members of CC1 possess an identical Ag type (Ag type I). MS types x and bb were found in samples with different Ag types (VII and VIII). Ag types VII and VIII differ by a single di-nucleotide indel at Ag marker SnSAG3, likely representative of drift rather than recombination as a mechanism to account for allele differences in this case. In contrast, MS type k has a markedly different Ag type (III) compared to other members of the CC2, which all possess Ag type II. Ag types II and III have different alleles at all Ag loci examined, making a recombination event the most parsimonious explanation for the difference between MS type k and other members of CC2 rather than genetic drift.

Since this and all previous studies of *S. neurona* have found a high level of sequence homology among strains [22]–[25], [47], we chose to analyze strain relatedness with the eBURST algorithm [48], [49]. This program helps eliminate confounding effects that low sequence diversity and moderate levels of recombination can have on other methods of intra-specific sequence analysis, such as clustering, dendrograms, and phylogenetic trees, as demonstrated in [22]–[24], by only focusing on single clones and their most recent descendents [48]–[50]. We adapted the MS data for the nine markers that permit simultaneous comparison of all strains (Sn2–Sn5, Sn7–Sn11) to serve as a multi-locus typing scheme. This typing scheme, which is based on the number of repeats at each locus, was amenable to use with this program. Using the default settings, which group isolates based on the premise that they are single locus variants (SLVs), or share 8 out of 9 alleles, we identified 8 clonal complexes (CC1–8), only 3 of which contained more than two genotypes, and 8 singletons (genotypes differing by 2 or more alleles from all others) (Table 1; Figure 3). Intriguingly, just two clonal complexes, CC1 and CC2, accounted for almost 64% (56/87) of the strains analyzed in this study (Figure 2C). This result held true even when correcting for bias introduced by the outbreak event by removing these samples from the data set, as 44/75 samples (59%) still belonged to CC1 or CC2.

All SLVs identified in this study differed by a single stepwise (i.e. a single di-nucleotide repeat) mutation, which supports the assumption that the eBURST groupings represent clonal complexes in which allelic variation is a result of mutation/drift and not recombination (Table S1) [50]. The only exceptions to this were SLVs ‘l’ and ‘o’, members of CC3, that differed by 3 di-nucleotide repeats at MS Sn11. These isolates were from a sea otter in California and a horse from Missouri so the greater number of stepwise mutations detected may be a result of extended geographic isolation, thus allowing time for more drift to occur (Table S1). A single mutation event that resulted in multiple stepwise mutations is also plausible.

Since recombination appeared to be rare between clonal complexes based on MS markers, we decided to overlay the results of the Ag typing analysis on the eBURST output (Figure 3). The results were consistent with previous claims of an intermediate population structure for *S. neurona* [22]–[25], [47] in that both clonal propagation and sexual recombination were supported. All members of CC1 and 29/30 members of CC2 possessed an identical Ag type (Ag types I and II, respectively). In contrast, all MS types in CC3 and CC8 possessed a distinct Ag type. There were also two cases (MS types ‘x’ and ‘bb’) where the same MS type was identified with two distinct Ag types (Ag types VII and VIII) and the reverse scenario also occurred where the same Ag type (VI) characterized three clonal complexes based on MS types (CC4, CC5, CC6), all of which could potentially indicate recombination events (Figure 3).

Overall, these data support a population structure that is highly clonal, though evidence for recombination is present as well. This intermediate population structure is similar to that described for *T. gondii*, though definitive conclusions will require a sample set less biased towards diseased animals [2]. It is worth noting here that the population structure of the organisms described in this study is, like all population genetic structures, only as resolved as the markers allow. For example, finer resolution can be achieved by applying the marker SnD2 from Rejmanek et al. [23] to SO4711, SO4786 and O7 to show that they are different strains. What this does not change, though, is that these strains are members of the same clonal complex and that resolution at this level is sufficient to identify an outbreak clone and to document geographic partitioning of strains along the California coastline (see below). This level of resolution is more robust to the possibility of strand slippage and evolution of new alleles during PCR that could make identical clones appear distinct with finer levels of resolution. An example of this may have occurred with SO4387, identified in this study as MS type ‘g,’ but by Rejmanek et al. [23] as MS type ‘i.’ These types differ by a single repeat at MS Sn9 (Table S1). It is also possible that this otter was co-infected with two closely related strains. Consistent identification of SLVs in many samples increases the confidence that they represent truly different strains. The outstanding potential these microsatellite markers have for more robust strain resolution, if interpreted cautiously, can facilitate addressing more specific questions, such as the identity and point source of an epidemic clone.

### Temporal stability, geographic distribution, and host distribution of strains in California

**Figure 4:**
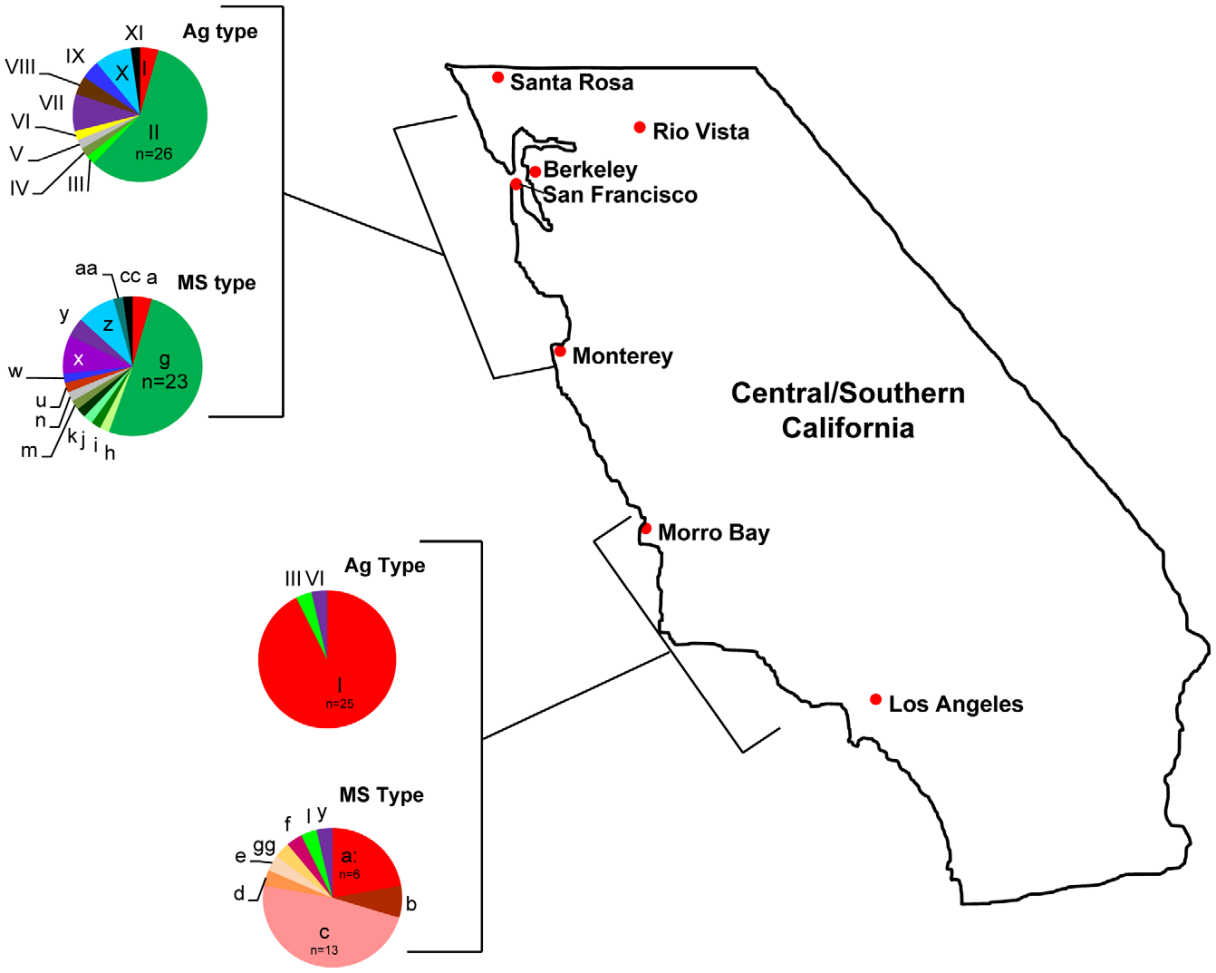
Geographic distribution of *Sarcocystis neurona* Ag and MS types in California. All sea otter samples were collected in two distinct, ∼200 km stretches along the California coast: one in central California from just north of San Francisco Bay to just south of Monterey Bay, and one to the south from just north of Morro Bay to just north of Los Angeles. Nearly all (93%) of the 27 samples from the southern region belonged to eBURST defined clonal complex (CC) 1 and none were identified as CC2. In the north, 63% of 45 samples belong to CC2 and only two representatives of CC1 were found. Terrestrial isolates from California were from 10 opossums and 4 horses. These, along with one sample from a porpoise, were from the northern range and included as such. The majority of sea otter samples were from two small areas of coastline: one near Monterey Bay in the north and the other near Morro Bay in the south (see Table S1 for details).

The majority of strains (72/87; 83%) evaluated in this study were collected from two distinct 200 km stretches along the California coast or the adjacent terrestrial environment (Table S1; Figure 4). As such, we utilized this subset of the data to examine the temporal stability of strains and their geographic and host distribution in central California.

**Figure 5:**
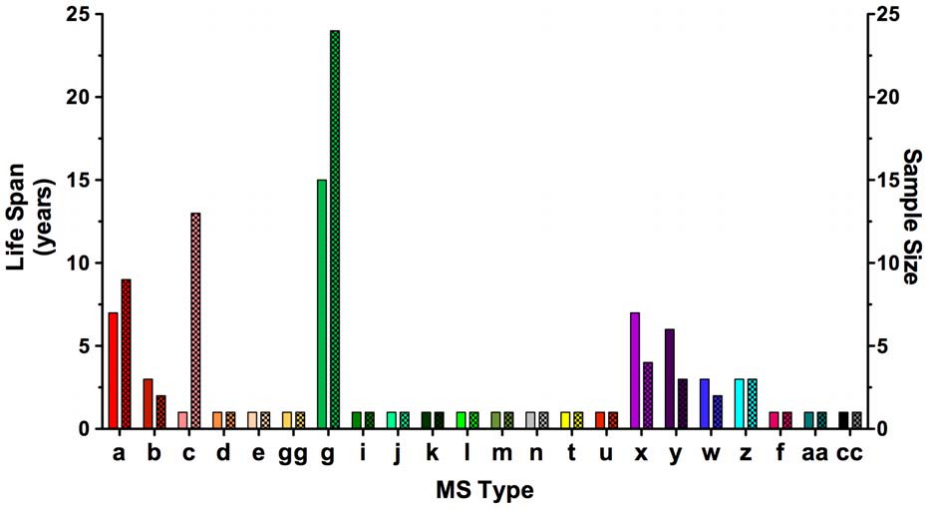
*Sarocystis neurona* MS type lifespan and sample size in California. Lifespans for microsatellite (MS) types (solid bars) were defined as the time period from identification the first representative sample to the last during the 15 years (1994–2009) encompassed in this study. Sample sizes are indicated by checkered bars. MS type ‘g,’ a member of eBURST defined clonal complex 2 (CC2), was the longest lived and most prevalent MS type, having a representative sample in all of the years for which samples were available. There were no *S. neurona* samples available for testing during 1996–1998. MS type ‘c,’ the genotype implicated in the sea otter epizootic, was only found in 2004 during the month of the outbreak and four months thereafter in the same region.

The total time period covered by the strains analyzed in this study is 15 years (1994–2009). Sample sizes were not evenly distributed across each year and some years (1996–1998) had no representative samples, so it is likely that genotype life spans are underestimated. Despite this, at least one clonal complex, CC2, appears to be very stable in nature over time, exhibiting a lifespan encompassing the entire length of this study. CC2 was sampled during 12 of the 13 years for which a sample was collected (Table S2). Within this complex, Ag type II, MS type ‘g’ had a lifespan of the full time period examined (15 years) and was the longest lived of any Ag or MS type (Figure 5; Table S2). The other clonal complexes present in California, CC2, CC3, CC6–CC8, appeared to be stable as well, with life spans ranging from 5–8 years (Table S2). Collectively these data provide supporting evidence for *S. neurona*'s ability to propagate clonally. However, it will be important to test whether or not these allelic combinations appear more often than would be expected by chance to confirm clonal propagation as more sequencing data becomes available from strains collected from non-diseased animals and the position of the markers in the genome is identified [51]. Interestingly, the genotype associated with the outbreak, Ag type I, MS type ‘c’, was only found during 2004 (Figure 5). These samples were all associated with otters dying during the epizootic in April, 2004, except for two samples that were obtained from sick otters in the same area four months after the event ended (Table S1). The implications these observations may have for strain virulence are discussed below.

**Figure 6:**
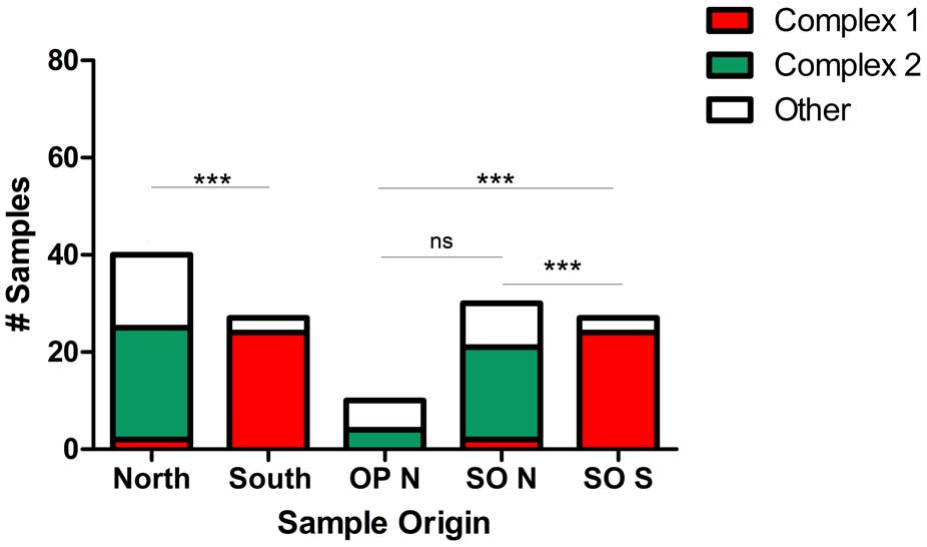
Geographic partitioning and host associations of *Sarcocystis neurona* strains. Distinct *S. neurona* populations as defined by the proportion of the population belonging to the dominant eBURST defined clonal complexes (CC) 1 or 2 were found infecting animals in the northern and southern ranges examined in California (see Figure 4). This difference remained significant by Chi-Square analysis when only sea otter samples were compared. When samples from sea otters from the northern range were compared to opossum samples from the adjacent terrestrial environment, no significant difference was found. There were no samples from terrestrial mammals in the southern range. OP N: opossum samples from the northern range; SO N: sea otter samples from the northern range; SO S: sea otter samples from the southern range; ns: not significant; ***p<0.00001.

On visual inspection, it appeared that the genetic composition of *S. neurona* strains from the Monterey Bay area was distinct from the southern strains obtained in or near Morro Bay (Figure 4; Table 1). We further tested this hypothesis by conducting χ^2^ analysis on the proportion of the majority clonal complexes (CC1 and CC2) that comprised each population. There was a highly significant difference between northern and southern strains (Figure 6). Significance remained when analysis was restricted to sea otter samples, in order to eliminate any confounding effects due to host species, because all southern strains were from sea otters (Figure 6). This conclusion is consistent with data reported previously on *S. neurona* strains from coastal California [24], [25], but contrasts with the conclusions of Rejmanek et al. [23].

We also sought to identify a potential terrestrial source for *S. neurona* strains present in the marine environment. Experimental evidence for the model organism, *T. gondii*, supports a route of infection for sea otters through ingestion of *S. neurona* sporocysts that were washed to the ocean in contaminated fresh water and then concentrated in the otters' filter-feeding invertebrate prey [52]–[54]. Implicating opossums as the ultimate terrestrial source of infection is supported by comparing the prevalence of the majority clonal complexes (CC1 and CC2) in sea otters and opossums in the northern, Monterey Bay area study site (the only locale from which opossum samples were obtained). Strain prevalence differences between these groups were not statistically different, suggesting that monitoring strain types in coastal dwelling opossums will be predictive of genotypes infecting adjacent marine dwelling otters (Figure 6). Observational data from the outbreak noting an abundance of razor clams and evidence of sea otter movement into the area for feeding (i.e. accumulation of broken shells on the shore) just prior to the event, further support this model of land-to-sea parasite transfer [33]. Sea otters very rarely consume known intermediate hosts of *S. neurona* [55], leaving the ingestion of sporocysts as the most biologically plausible route for sea otter infection regardless of the land-to-sea transport mechanism, and strongly supporting the conclusion that this outbreak originated from a selfing event in the opossum host.

### Parasite genotypes and virulence

Disease is a complex manifestation of the interplay between intrinsic pathogen factors (i.e. pathogen genotype) and numerous external factors, including dose, host immune status, and environmental conditions such as weather that can influence transmission. Delineating the relative contribution of each of these factors to a given disease outbreak is a difficult process, as is illustrated by the outbreaks described in this study. It is plausible that the *S. neurona* strain associated with the 2004 epizootic is intrinsically more virulent than other strains since it was only identified during the time period surrounding the outbreak and may have been too virulent for continued propagation. Also, the majority of otters infected died within 24–48 hours of stranding and had high IgM titers [33]. The rapid rise and subsequent fall of a virulent strain type is a phenomenon noted in many outbreaks of a diverse array of pathogens from viruses (e.g. Influenza virus [3]) to bacteria (e.g. *Leptospira interrogans* [56]) to fungi (e.g. *Coccidioides immitis* [57]). However, this phenomenon may also be attributable to sampling biases [2] or environmental factors [57] making the assumption that the virulent genotype is not adaptive inaccurate. Equally in the case of the sea otter outbreak, numerous external factors, including concurrent infection with other pathogens and domoic acid poisoning, abundant food source with potential for contamination with sporocysts, and a large rainstorm preceding the event that could have increased sporocyst deposition, may have played a contributing role in conferring this *S. neurona* strain with a virulent phenotype [33].

Similarly, the *T. gondii* strain implicated in the 2001 Brazil outbreak appeared to rise in prevalence during the outbreak but then decline over time in the local environment [44]. This was also a unique, newly identified genotype that caused symptomatic disease in 155 immune-competent individuals—an unusual phenomenon for this normally asymptomatic parasite. Importantly, though, ∼270 other individuals with access to the same water cistern seroconverted during this time with no overt signs of disease [44], invoking a role for environmental and host factors in this outbreak.

A striking character of both these outbreak events is the key role self-mating in the definitive host served as a catalyst allowing virulent pathogen genotypes to rapidly reach high levels under the right conditions to precipitate a disease epidemic.

### Self-mating potentiated the emergence of the *S. neurona* and *T. gondii* epidemic clones

Epidemic clonality associated with sporocyst or oocyst ingestion strongly suggests that self-mating in the definitive host was the key event leading to these outbreaks. Selfing in the definitive host has been confirmed experimentally for *T. gondii* [31], [32] but only indirectly assumed for *S. neurona* [58]. Prior to this study, rigorous genetic characterization of selfing events in nature were lacking and the question as to whether a productive sexual out-cross or a selfing event precedes an outbreak linked to oocysts or sporocysts had not previously been tested.

Early population genetic studies using limited, poorly resolved markers identified a paucity of mixed strain *T. gondii* or *S. neurona* infections in nature and these data have previously been interpreted to suggest that most definitive host infections would be by a single strain and therefore out-crossing would be rare in nature [59]. However, more recent studies using unbiased, multi-locus typing schemes have consistently identified mixed strain infections among natural intermediate hosts suggesting that prey species of definitive hosts are more frequently harboring mixed strain infections than previously envisaged [60]–[72]. Hence, the lack of mixed strain infections identified in earlier studies may simply reflect the techniques used, such as bioassay or limited genetic typing, that were biased toward certain strains and likely missed multiple infections and the true diversity of genotypes present.

As more high resolution, multilocus genetic markers are being applied against previously characterized strains of *T. gondii*, an increasing number are being re-classified as recombinants, defined as products of sexual out-crossing events, including strains previously linked to outbreaks [20]. Given the virulent nature of the two outbreaks examined here, and the evidence that out-crossing between two avirulent, haploid parents can produce progeny with enhanced virulence [14], we originally hypothesized that out-crossing might explain the genetic origin and expansion of the outbreak strains, rather than self-mating. Intriguingly, close examination of the environmental isolates surrounding the *T. gondii* outbreak supported this hypothesis because the epidemic clone was one of many progeny produced by a local genetic out-cross. However, the available evidence indicated that, while out-crossing certainly preceded the outbreak, it was the subsequent selfing event that was responsible for the epidemic expansion and transmission of the virulent clone that caused the outbreak. Certainly this dataset argues that sex and self-mating combined to produce the *T. gondii* clonal outbreak. Further typing of additional outbreaks is warranted to examine whether or not an out-cross is independently sufficient to cause an epidemic attributable to multiple, recombinant progeny.

This two-step process of local epidemic expansion via a sexual out-cross followed by clonal propagation of a few progeny with enhanced adaptations or virulence is reminiscent of the process envisioned on a larger scale for the pandemic rise of the archetypal *T. gondii* clones (Types I, II, and III), also found to be the progeny of an out-cross [13], [14]. Documenting this process in real time at a local level has provided key insight into mechanisms that account for clonal propagation in nature. It was previously proposed based on laboratory studies that clonal dominance of archetypal *T. gondii* strains was attributable to an enhanced ability for oral transmission through carnivory, a hypothesis which certainly warrants further investigation in natural settings [19]. However, recent studies have since shown that this trait does not operate as originally proposed [73], [74]. These findings raised the possibility that other life history traits may likewise be important in perpetuating clones.

In this light, it is worth noting that all aspects of the parasite lifecycle that promote clonal propagation, namely selfing, oral transmission through carnivory, and transplacental transmission, contribute in part to clonality in the population structure. However, when considering their relative roles, the advantage in fecundity the sexual stage can impart during a selfing event to a single parasite genotype, as documented in this study, provides strong evidence this mechanism is likely the major contributor to localized or regional clonal dominance of certain strains. The basic reproductive number (R_0_), or number of secondary infections a single infected individual will cause, is many orders of magnitude greater in the definitive host (which releases millions of environmentally stable, infectious propagules capable of waterborne or aerosolized transmission [75]) compared to an intermediate host (in which the infectious units produced can only be passed to those directly feeding on tissues). Oocysts or sporocysts can also successfully infect intermediate hosts at much lower doses (even a single oocyst) than tissue cysts [76], [77]. Oocyst deposition therefore exists as a potent mechanism for causing widespread epidemics and establishes a plausible rationale for explaining how selective sweeps can occur among these heterogamous pathogens. Determining what factors govern whether these sweeps occur on a local, and presumably more frequent, epidemic level or reach pandemic proportions are important subjects for future research.

Our results also confirm that fecal contamination of food and water sources represents a major threat to human and animal health, hence targeting the definitive host or the oocyst stage of these parasites is an excellent first-step strategy to disrupt transmission. This conclusion is further supported by studies showing the importance of the definitive host stage for maintaining continued transmission of this parasite in island communities [34]–[37] and how local vaccination of definitive feline hosts can significantly reduce *T. gondii* infection rates [78].

The scope of explanatory power for this selfing model can also be extended to other highly clonal, cyst forming parasites, including the clonal outbreak linked to *S. neurona* and likely other pathogenic *Sarcocystis spp.* and *Neospora spp*. This finding is significant since many aspects of the *T. gondii* life cycle have previously been proposed to be unique to this species among the tissue encysting coccidia, including its broad host range inclusive of nearly all warm-blooded vertebrates and its ability to be transmitted through carnivory among intermediate hosts [19]–[21,] (but also see: [79], [80]–[83]). Notably, selfing has also been demonstrated in more distantly related Apicomplexan parasites, including *Eimeria spp.* and *Plasmodium spp*. [31]. In addition, the processes of homothalism and same-sex mating identified in fungi serve the analogous purpose of clonal propagation via a mechanism more generally thought to serve in genetic recombination and out-crossing [84]. This suggests that selfing, as a genetic mechanism of clonal propagation, has potential to play a pivotal and previously under-recognized role for a diverse array of eukaryotic pathogens in the expansion of genotypes that cause disease epidemics and/or emerge as highly successful clonotypes to rapidly alter population genetic structures.

## Materials and Methods

### Ethics statement

Work in California was conducted under United States Fish and Wildlife Service (USFWS) permit MA 491 672724-9 issued to United States Geological Survey Biological Resource Discipline (USGS492 BRD). Harbor seal carcasses were gathered and samples processed as part of Northwest Marine Mammal Stranding Network activities authorized under Marine Mammal Protection Act (MMPA) Stranding Agreements (SA), and Section 109(h) (16 U.S.C. 1379(h)). Additional specimens were acquired under MMPA Section 120, and the National Marine Fisheries Service (NMFS) MMPA Research Permit 782–1702.

### *Sarcocystis neurona* and *Toxoplasma gondii* DNA and genetic typing markers

Parasite DNA was obtained either from infected host tissues or parasite isolates maintained in tissue culture as described previously [25]. Samples were analyzed using a typing scheme that included the surface antigen markers: SnSAG1, SnSAG3, SnSAG4, SnSAG5, SnSAG6 [25] and 9 microsatellite markers Sn2–Sn5 and Sn7–Sn11 originally described by Asmundsson and Rosenthal [85] but applied as modified in Wendte et al. [25] and Rejmanek et al. [23]. Three additional microsatellite markers were designed by the following method: Publically available *Sarcocystis neurona* expressed sequence tags (ESTs) were downloaded from the NCBI dbEST database (http://www.ncbi.nlm.nih.gov/dbEST) and the *S. neurona* Gene Index (maintained by the Computational Biology and Functional Genomics Laboratory at the Dana Farber Cancer Institute, http://compbio.dfci.harvard.edu/tgi/) databases. The downloaded ESTs were assembled into contigs using the SeqMan (Lasergene) application. Contig sequences were then processed with the MISA microsatellite identification program (http://pgrc.ipk-gatersleben.de/misa/) with the following repeat parameters: definition (unit size-minimum repeats): 2-12, 3-7, 4-5, 5-4, 6-3, 7-3, 8-2, 9-2, 10-2, 11-2, 12-2, 13-2, 14-2, 15-2; interruptions (maximum difference between 2 simple sequence repeats): 25.

Approximately 50 microsatellites of sufficient length and/or complexity were identified. Three (Sn1520, Sn1863 and Sn515) of these markers were not previously published and possessed sufficient non-redundant flanking sequence to allow for nested primer design and produced robust size-polymorphic PCR amplification products. Primers were validated as described [25] and found to be specific and sensitive for *S. neurona* DNA in tissues (data not shown). The primers designed are as follows: Sn1520 Fext- GGGGCAGAACCATCGTAGTA, Rext- GTGAAGCATTTCCCCTACGA, Fint- GGCGGTAGTCACTTGCTGA, Rint- GTGGGAGAAGACGGTCGTTA; Sn1863 Fext- CATGGCGTGCGTTAACTAAA, Rext- CGTACAAACACACGCTCCAC, Fint- CCATTCATCGACAGCGACTA, Rint- TGAGACAGCCGTCAAACACT; Sn515 Fext- CTTCTAGCGGCTGTTTCTCC, Rext- TCTGTGTGGGTGTGGAAGTC, Fint- GACCCCCTCTCTGCTTCTCT, Rint- ACGCAAATGCGAACATATCA. Representative sequences for each allele at each locus were placed in GenBank under the following accession numbers: Sn1520: HM851251, HM851252, HM851253, HM851254, HM851255; Sn1863: HM851256, HM851257, HM851258, HM851259; Sn515: HM851249, HM851250. PCR, DNA sequencing and analysis were conducted as described previously, except, to control for bias in scoring results, random sample IDs were assigned to samples before sequencing so that sequence analysis for some loci was blinded [25].

For this study, *S. neurona* DNA from 15 sea otters and 4 harbor seals was analyzed. Additionally, samples from 21 sea otters, 2 harbor seals, 3 horses, and 2 raccoons previously described by Wendte et al. [25] at the SnSAG antigen loci and MS Sn9, were further typed in this study at the remaining 10 MS loci. Finally, *S. neurona* DNA from 21 sea otters, 1 porpoise, 4 horses, 13 opossums, and 1 cat that was previously typed by Rejmanek et al. [23] at SnSAG3, SnSAG4, and MS markers Sn2–Sn5 and Sn7–Sn11 were combined with the data in this study for a total sample set that included 87 samples from 57 sea otters, 6 harbor seals, 2 raccoons, 13 opossums, 7 horses, 1 porpoise, and 1 cat. In all, 75 of the 87 samples were from California. Other states represented include Georgia (n = 2 samples), Illinois (n = 1), Missouri (n = 3), Washington (n = 4), and Wisconsin (n = 2). Some overlap existed between the samples typed in this study and those reported by Rejmanek et al.: samples SO4387, SO4413, H1, H2, and H3 in this study are reported as SO1, SO2, Horse 1, Horse 2, and Horse 3 in Rejmanek et al. [23], respectively. Complete information about the sample origins is found in Table S1.

*Toxoplasma gondii* isolates from a water cistern (n = 2), chickens (n = 11), and one cat associated with a human waterborne toxoplasmosis outbreak [44], as well as laboratory strain CEP were typed at microsatellite loci B17, B18, TgMA, TUB2, W35 [39] and M95 [40]. Markers were PCR amplified and sequenced to assign alleles as for *S. neurona* markers [25]. Representatives of each microsatellite allele at each locus were placed in Genbank under accession numbers: B17: HM851260–67; TgMA: HM851268–73; W35: HM851274–77; M95: HM851278–81.

### Genotyping and eBURST analysis

Because different parts of the genome are likely under different selective pressures, all *S. neurona* samples were categorized by an antigen (Ag) type designated by roman numerals and a microsatellite (MS) type indicated by a lowercase letter designation. Ag types were defined by the presence/absence of mutually exclusive antigen genes (SnSAG1, SnSAG5, or SnSAG6) and the inheritance pattern of alleles at SnSAG3 and SnSAG4 [23], [25]. MS types were assigned on the basis of allele combinations defined by the number of di- or tri- nucleotide repeats at each locus (Sn2–Sn5 and Sn7–Sn11, Sn1520, Sn1863). Sn515 was a complex repeat in which each isolate possessed one of two alleles. Samples from the study by Rejmanek et al. [23] were not typed at the SnSAG1-5-6 loci, but were placed into Ag groups based on the allelic profile at SnSAG3 and SnSAG4 and by the Ag group their MS type was associated with in samples typed at all markers. For example, based on the alleles at SnSAG3 and SnSAG4, sample SO4 (Table S1) could be placed either in Ag type II or V, but its MS type was only found associated with Ag type II in samples where all markers were typed, making this the most likely, though not definitive, Ag type designation. The *S. neurona* strains assessed by Rejmanek et al. [23] were also not typed at MS markers Sn1520, Sn1863, and Sn515. Presumptively classifying these samples into MS types based on alleles at Sn2–Sn5 and Sn7–Sn11 is likely accurate, though, since these three markers did not provide additional resolution to MS types for the 46 additional *S. neurona* strains described in this study.

The alleles present at MS markers Sn2–Sn5 and Sn7–Sn11 were used for creation of a multi-locus sequence typing scheme by which all isolates could be compared. The numerical designation of alleles allowed the detection of which MS types formed clonal complexes using the eBURST program [48]. Default settings were used which grouped MS types on the basis of sharing alleles at 8 of the 9 markers analyzed.

To assess *T. gondii* isolates for clonality, MS alleles were combined with previously published DNA sequence analysis at three genetic loci, PCR-RFLP or DNA sequencing at 10 loci, and serologic analysis as described by Vaudaux et al. [44].

### Statistical analysis

Statistical analyses were performed using GraphPad Prism 5 and χ^2^ values were considered significant at P = 0.05.

## Supporting Information

Table S1 *Sarcocystis neurona* genotypes and sample source information.
(0.02 MB PDF)

Table S2 *Sarcocystis neurona* genotype presence over time in California.
(0.01 MB PDF)
