# Supplementary material for: Self-Mating in the Definitive Host Potentiates Clonal Outbreaks of the Apicomplexan Parasites Sarcocystis neurona and Toxoplasma gondii
Source: PLoS Genet. 2010 Dec 23;6(12):e1001261. doi: 10.1371/journal.pgen.1001261 (PMC3009688; doi:10.1371/journal.pgen.1001261)
Supplement: Table S1 — Sarcocystis neurona genotypes and sample source information. (0.02 MB PDF) [file pgen.1001261.s001.pdf]

Table S1

Sarcocystis neurona genotypes and sample source information‡

						Genetic Marker																	
						SnSAG1-5-6	SnSAG3*				SnSAG4†	Sn2 (GT)n	Sn3 (AT)n	Sn4 (CA)n	Sn5 (CA)n	Sn7 (CA)n	Sn8 (CA)n	Sn9 (GT)n	Sn10 (AT)n	Sn11 (CA)n	Sn1520 (CTA)n	Sn1863 (AC)n	Sn515 complex
							239	503	504	1057	592												
Sample	ATOS/Geo. Loc.	Date collected	Source#	Ag Type	MS Type		C	A	T	C	C												NT count
SO3106	318	Apr-99	Wendte et al.	II	i	1	G	·	·	T	·	9	12	12	9	17	10	17	11	13	9	17	120
SO3339	46	Apr-00	This study	V	n	5	G	·	·	T	·	9	11	12	9	18	10	16	9	13	9	15	81
SO3483	820	Mar-01	Wendte et al.	I	a	5	·	·	·	·	·	10	11	13	9	22	10	17	9	14	10	16	81
SO3485	827	Mar-01	Wendte et al.	I	a	5	·	·	·	·	·	10	11	13	9	22	10	17	9	14	10	16	81
SO3501	915	Apr-01	Wendte et al.	I	a	5	·	·	·	·	·	10	11	13	9	22	10	17	9	14	10	16	81
SO3508	827	Apr-01	This study	I	b	5	·	·	·	·	·	10	11	13	9	21	10	17	9	14	10	16	81
SO3523	141	May-01	Wendte et al.	VII	y	6	·	·	·	·	G	9	11	13	9	19	10	14	9	15	10	14	81
SO3528	292	May-01	Wendte et al.	II	g	1	G	·	·	T	·	9	12	12	9	17	10	18	11	13	9	17	120
SO3629	276	Dec-01	Wendte et al.	II	g	1	G	·	·	T	·	9	12	12	9	17	10	18	11	13	9	17	120
SO3634	258	2001	This study	X	z	6	·	·	·	·	·	9	10	13	10	20	10	14	9	14	10	14	81
SO3639	299	Dec-01	Wendte et al.	VII	x	6	·	·	·	·	G	9	11	13	9	20	10	14	9	15	10	14	81
SO3660	384	2002	This study	II	g	1	G	·	·	T	·	9	12	12	9	17	10	18	11	13	9	17	120
SO3866	309	Apr-03	Wendte et al.	II	g	1	G	·	·	T	·	9	12	12	9	17	10	18	11	13	9	17	120
SO3892	917	Apr-03	Wendte et al.	I	a	5	·	·	·	·	·	10	11	13	9	22	10	17	9	14	10	16	81
SO4135	824	Mar-04	Wendte et al.	I	c	5	·	·	·	·	·	10	11	13	9	21	10	17	9	13	10	16	81
SO4151	825	Apr-04	Wendte et al.	I	c	5	·	·	·	·	·	10	11	13	9	21	10	17	9	13	10	16	81
SO4166	827	Apr-04	Wendte et al.	I	c	5	·	·	·	·	·	10	11	13	9	21	10	17	9	13	10	16	81
SO4167	827	Apr-04	Wendte et al.	I	c	5	·	·	·	·	·	10	11	13	9	21	10	17	9	13	10	16	81
SO4168	832	Apr-04	Wendte et al.	I	d	5	·	·	·	·	·	10	11	12	9	21	10	17	9	13	10	16	81
SO4169	819	Apr-04	Wendte et al.	I	c	5	·	·	·	·	·	10	11	13	9	21	10	17	9	13	10	16	81
SO4171	819	Apr-04	Wendte et al.	I	c	5	·	·	·	·	·	10	11	13	9	21	10	17	9	13	10	16	81
SO4177	925	Apr-04	This study	I	c	5	·	·	·	·	·	10	11	13	9	21	10	17	9	13	10	16	81
SO4183	919	Apr-04	This study	I	c	5	·	·	·	·	·	10	11	13	9	21	10	17	9	13	10	16	81
SO4189	808	Apr-04	This study	I	c	5	·	·	·	·	·	10	11	13	9	21	10	17	9	13	10	16	81
SO4195	818	Apr-04	This study	I	c	5	·	·	·	·	·	10	11	13	9	21	10	17	9	13	10	16	81
SO4202	925	Apr-04	This study	I	c	5	·	·	·	·	·	10	11	13	9	21	10	17	9	13	10	16	81
SO4174	1137	Apr-04	This study	I	b	5	·	·	·	·	·	10	11	13	9	21	10	17	9	14	10	16	81
SO4178	1135	Apr-04	Wendte et al.	I	f	5	·	·	·	·	·	10	11	13	9	22	10	18	9	13	11	16	81
SO4181	303	Apr-04	Wendte et al.	II	g	1	G	·	·	T	·	9	12	12	9	17	10	18	11	13	9	17	120
SO4194	21	Apr-04	Wendte et al.	IX	w	1	·	·	·	·	·	9	10	13	9	20	10	14	9	14	10	14	81
SO4240	261	Jun-04	This study	X	z	6	·	·	·	·	·	9	10	13	10	20	10	14	9	14	10	14	81
SO4285	806	Aug-04	Wendte et al.	I	c	5	·	·	·	·	·	10	11	13	9	21	10	17	9	13	10	16	81
SO4289	827	Aug-04	This study	I	c	5	·	·	·	·	·	10	11	13	9	21	10	17	9	13	10	16	81
SO4413	920	Feb-05	This study	III	l	5	G	·	·	T	G	9	11	12	9	18	10	17	10	13	10	15	81
SO4529	396	Jul-05	This study	II/V	g	na	G	·	·	T	·	9	12	12	9	17	10	18	11	13	9	17	120
SO4387	337	2005	This study	II	g	1	G	·	·	T	·	9	12	12	9	17	10	18	11	13	9	17	120
SO4530	924	2005	Rejmanek et al.	I	e	na	·	·	·	·	·	10	11	12	9	21	10	16	9	14			

Table S1 Continued

Sarcocystis neurona genotypes and sample source information

						Genetic Marker																	
Sample	ATOS/Geo. Loc.	Date collected	Source	Ag Type	MS Type	SnSAG1-5-6	SnSAG3* 239 C	503 A	504 T	1057 C	SnSAG4† 592 C	Sn2 (GT)n	Sn3 (AT)n	Sn4 (CA)n	Sn5 (CA)n	Sn7 (CA)n	Sn8 (CA)n	Sn9 (GT)n	Sn10 (AT)n	Sn11 (CA)n	Sn1520 (CTA)n	Sn1863 (AC)n	Sn515 complex NT count
SO4653	258	2006	Rejmanek et al.	II/V	g	na	G	·	·	T	·	9	12	12	9	17	10	18	11	13			
SO4697	321	2006	Rejmanek et al.	II/V	g	na	G	·	·	T	·	9	12	12	9	17	10	18	11	13			
SO4711	321	2006	Rejmanek et al.	II/V	g	na	G	·	·	T	·	9	12	12	9	17	10	18	11	13			
SO4725	321	2006	Rejmanek et al.	VI/VII	u	na	·	·	·	·	G	10	10	13	9	18	10	14	9	14			
SO4755	808	2006	Rejmanek et al.	I	a	na	·	·	·	·	·	10	11	13	9	22	10	17	9	14			
SO4786	370	2006	Rejmanek et al.	II/V	g	na	G	·	·	T	·	9	12	12	9	17	10	18	11	13			
SO4834	260	2006	Rejmanek et al.	II/V	h	na	G	·	·	T	·	9	12	12	9	16	10	18	11	13			
SO4928	260	2007	Rejmanek et al.	II/V	g	na	G	·	·	T	·	9	12	12	9	17	10	18	11	13			
SO4970	260	2007	Rejmanek et al.	II/V	g	na	G	·	·	T	·	9	12	12	9	17	10	18	11	13			
SO4972	812	2007	Rejmanek et al.	VI/VII	y	na	·	·	·	·	G	9	11	13	9	19	10	14	9	15			
SO5002	259	2007	Rejmanek et al.	VI/VII	y	na	·	·	·	·	G	9	11	13	9	19	10	14	9	15			
SO5073	261	2007	Rejmanek et al.	II/V	g	na	G	·	·	T	·	9	12	12	9	17	10	18	11	13			
SO5110	323	2007	Rejmanek et al.	II/V	g	na	G	·	·	T	·	9	12	12	9	17	10	18	11	13			
SO5226	289	2008	Rejmanek et al.	III	k	na	G	·	·	T	G	9	13	12	9	17	10	18	11	13			
SO5259	827	2008	Rejmanek et al.	I	a	na	·	·	·	·	·	10	11	13	9	22	10	17	9	14			
SO5263	833	2008	Rejmanek et al.	I	gg	na	·	·	·	·	·	10	11	12	9	21	10	17	9	14			
SO5274	321	2008	Rejmanek et al.	I	a	na	·	·	·	·	·	10	11	13	9	22	10	17	9	14			
SO5278	321	2008	Rejmanek et al.	I	a	na	·	·	·	·	·	10	11	13	9	22	10	17	9	14			
SO5283	330	2008	Rejmanek et al.	IV	m	na	G	-	-	T	·	9	11	12	9	18	10	17	9	13			
SO5296	318	2008	Rejmanek et al.	II/V	g	na	G	·	·	T	·	9	12	12	9	17	10	18	11	13			
HS1423	CA	Jul-99	Wendte et al.	VI	t	5	·	·	·	·	G	10	10	13	9	17	10	14	9	14	9	15	81
HS1531	CA	Jul-99	Wendte et al.	II	g	1	G	·	·	T	·	9	12	12	9	17	10	18	11	13	9	17	120
HS0604	WA	Jun-04	This study	VI	r	5	·	·	·	·	G	10	11	13	9	18	10	14	10	13	14	16	81
HS0604-03	WA	Jun-04	This study	?	ee	na	na	na	na	na	na	9	10	15	9	19	10	16	8	14	na	16	81
HS0606-06	WA	Jun-06	This study	VI/VII	s	na	·	·	·	·	G	10	11	13	9	19	10	16	10	14	14	16	81
HSGI07-12	WA	2007	This study	VI	ff	5	·	·	·	·	G	10	11	13	9	19	10	16	10	13	14	16	81
Porp	Monterey, CA	2006	Rejmanek et al.	II/V	g	na	G	·	·	T	·	9	12	12	9	17	10	18	11	13			
H1	Santa Rosa, CA	1994	Wendte et al.	II	g	1	G	·	·	T	·	9	12	12	9	17	10	18	11	13	9	17	
H2	Santa Rosa, CA	1994	Wendte et al.	II	g	1	G	·	·	T	·	9	12	12	9	17	10	18	11	13	9	17	
H3	CA	1995	Wendte et al.	II	g	1	G	·	·	T	·	9	12	12	9	17	10	18	11	13	9	17	120
H4	MO	1999	Rejmanek et al.	XII	dd	na	·	·	·	G	G	10	16	13	9	19	11	14	10	14			
H5	MO	1999	Rejmanek et al.	I/IX/X/?	o	na	·	·	·	·	·	9	11	12	9	18	10	17	10	16			
H6	Berkeley, CA	2009	Rejmanek et al.	II/V	g	na	G	·	·	T	·	9	12	12	9	17	10	18	11	13			
H7	Berkeley, CA	2009	Rejmanek et al.	XI	cc	na	·	-	-	·	G	9	13	13	10	16	10	14	9	14			
O1	Monterey, CA	2005	Rejmanek et al.	X	z	na	·	·	·	·	·	9	10	13	10	20	10	14	9	14			
O2	Monterey, CA	2006	Rejmanek et al.	X	z	na	·	·	·	·	·	9	10	13	10	20	10	14	9	14			
O3	Monterey, CA	2005	Rejmanek et al.	VIII	x	na	·	-	-	·	G	9	11	13	9	20	10	14	9	15			
O4	Rio vista, CA	2007	Rejmanek et al.	II/V	j	na	G	·	·	T	·	9	12	12	9	17	10	18	10	13			

Table S1 Continued

Sarcocystis neurona genotypes and sample source information

						Genetic Marker																	
						SnSAG1-5-6	SnSAG3*				SnSAG4†	Sn2 (GT)n	Sn3 (AT)n	Sn4 (CA)n	Sn5 (CA)n	Sn7 (CA)n	Sn8 (CA)n	Sn9 (GT)n	Sn10 (AT)n	Sn11 (CA)n	Sn1520 (CTA)n	Sn1863 (AC)n	Sn515 complex
							239	503	504	1057	592												
Sample	ATOS/Geo. Loc.	Date collected	Source	Ag Type	MS Type		C	A	T	C	C												NT count
O5	Monterey, CA	2007	Rejmanek et al.	II/V	g	na	G	·	·	T	·	9	12	12	9	17	10	18	11	13			
O6	Monterey, CA	2008	Rejmanek et al.	VI/VII	x	na	·	·	·	·	G	9	11	13	9	20	10	14	9	15			
O7	Monterey, CA	2008	Rejmanek et al.	II/V	g	na	G	·	·	T	·	9	12	12	9	17	10	18	11	13			
O8	Monterey, CA	2008	Rejmanek et al.	VIII	x	na	·	-	-	·	G	9	11	13	9	20	10	14	9	15			
O9	Monterey, CA	2008	Rejmanek et al.	II/V	g	na	G	·	·	T	·	9	12	12	9	17	10	18	11	13			
O10	Rio vista, CA	2007	Rejmanek et al.	IX	aa	na	·	·	·	·	·	9	10	13	9	17	10	18	9	13			
O11	GA	2008	Rejmanek et al.	VI/VII	bb	na	·	·	·	·	G	10	12	13	9	23	10	14	11	14			
O12	GA	2008	Rejmanek et al.	VIII	bb	na	·	-	-	·	G	10	12	13	9	23	10	14	11	14			
O13	IL	2008	Rejmanek et al.	VI/VII	v	na	·	·	·	·	G	10	11	13	10	16	10	14	10	14			
R1	WI	2006	Wendte et al.	VI	q	5	·	·	·	·	G	10	11	14	9	17	10	14	10	14	15	16	81
R2	WI	2006	Wendte et al.	VI	p	5	·	·	·	·	G	10	11	13	9	17	10	14	10	14	15	16	81
Cat	MO	2000	Rejmanek et al.	II/V	g	na	G	·	·	T	·	9	12	12	9	17	10	18	11	13			

ATOS: The 'As The Otter Swims' number refers to each sea otter's stranding location, based upon defined and sequential 0.5 kilometer segments of the California coastline, starting with zero (0) just north of San Francisco and increasing numerically from north to south.

SO: Sea Otter; HS: Harbor Seal; Porp: Porpoise; H: Horse; O: Opossum; R: Raccoon

CA: California; WA: Washington; MO: Missouri; GA: Georgia; IL: Illinois; WI: Wisconsin

NT: nucleotide

na: Not Available

*numbers refer to nucleotide position on reference sequence: GQ851954

†numbers refer to nucleotide position on reference sequence: GQ851957

‡Samples are organized and color coded by host species in the left columns (Blue: sea otter; Dark Blue: sea otters stranded in the 2004 epizootic; Sea Green: marine mammals other than sea otters; Brown: terrestrial mammals) and then color coded by antigen (Ag) and Microsatellite (MS) type in the remaining columns

#Samples from Wendte et al. were additionally typed at MS markers Sn2-Sn5, Sn7, Sn8, Sn10, Sn11, Sn1520, Sn1863, Sn515 for the current study
