# Supplementary material for: Self-Mating in the Definitive Host Potentiates Clonal Outbreaks of the Apicomplexan Parasites Sarcocystis neurona and Toxoplasma gondii
Source: PLoS Genet. 2010 Dec 23;6(12):e1001261. doi: 10.1371/journal.pgen.1001261 (PMC3009688; doi:10.1371/journal.pgen.1001261)
Supplement: Table S2 — Sarcocystis neurona genotype presence over time in California. (0.01 MB PDF) [file pgen.1001261.s002.pdf]

Table S2 Genotype Presence Over Time in California

			Year															
Clonal Complex	Ag type	MS type	94	95	99	00	01	02	03	04	05	06	07	08	09	Total	ST Life span (years)	CC Life span (years)
1	I	a					3		1			1		3		8	7	7
1	I	b					1			1						2	3	
1	I	c								13						13	1	
1	I	d								1						1	1	
1	I	e									1					1	1	
1	I	gg												1		1	1	
2	II	g	2	1			2	1	1	1	2	6	5	3	1	24	15	15
2	II	i			1											1	1	
2	II	j											1			1	1	
2	III	k												1		1	1	
3	III	l									1					1	1	8
3	IV	m												1		1	1	
3	V	n				1										1	1	
6	VI	t			1											1	1	7
6	VI	u										1				1	1	
7	VII	x					1							1		2	7	7
7	VII	y					1						2			3	6	
7	VIII	x									1			1		2	3	
8	IX	w					1			1						2	3	5
8	X	z								1	1	1				3	3	
Singleton	I	f								1						1	1	
Singleton	IX	aa											1			1	1	
Singleton	XI	cc													1	1	1	
	Total		2	1	2	1	9	1	2	19	6	9	9	11	2			

Ag: Antigen gene

MS: Microsatellite

ST: Sequence Type (combination of Ag and MS type)

CC: Clonal Complex
